# A Novel Remote Follow-Up Tool Based on an Instant Messaging/Social Media App for the Management of Patients With Low Anterior Resection Syndrome: Pilot Prospective Self-Control Study

**DOI:** 10.2196/22647

**Published:** 2021-03-19

**Authors:** Fan Liu, Peng Guo, Xiangqian Su, Ming Cui, Jianlong Jiang, Suo Wang, Zhouman Yu, Runhe Zhou, Yingjiang Ye

**Affiliations:** 1 Department of Gastroenterological Surgery Peking University People's Hospital Beijing China; 2 Beijing Key Laboratory of Colorectal Cancer Diagnosis and Treatment Research Beijing China; 3 Key Laboratory of Carcinogenesis and Translational Research (Ministry of Education), Department of Gastrointestinal Surgery IV Peking University Cancer Hospital and Institute Beijing China; 4 Department of General Surgery Changshu Hospital Affiliated to Soochow University, First People's Hospital of Changshu City Changshu China; 5 Department of General Surgery QiLu Hospital (Qingdao), Cheeloo College of Medicine Shandong University Qingdao China

**Keywords:** instant messaging social media, rectal cancer, low anterior resection syndrome, follow-up, telephone interview

## Abstract

**Background:**

Low anterior resection syndrome (LARS) is a common functional disorder that develops after patients with rectal cancer undergo anal preservation surgery. Common approaches to assess the symptoms of patients with LARS are often complex and time-consuming. Instant messaging/social media has great application potential in LARS follow-up, but has been underdeveloped.

**Objective:**

The aim of this study was to compare data between a novel instant messaging/social media follow-up system and a telephone interview in patients with LARS and to analyze the consistency of the instant messaging/social media platform.

**Methods:**

Patients with R0 resectable rectal cancer who accepted several defecation function visits via the instant messaging/social media platform and agreed to a telephone interview after the operation using the same questionnaire including subjective questions and LARS scores were included. Differences between the 2 methods were analyzed in pairs and the diagnostic consistency of instant messaging/social media was calculated based on telephone interview results.

**Results:**

In total, 21 questionnaires from 15 patients were included. The positive rates of defecation dissatisfaction, life restriction, and medication use were 10/21 (48%), 11/21 (52%), and 8/21 (38%) for telephone interview and 10/21 (48%), 13/21 (62%), and 5/21 (24%) for instant messaging/social media, respectively. No statistically significant difference was observed between instant messaging/social media and telephone interview in terms of total LARS score (mean 22.4 [SD 11.9] vs mean 24.7 [SD 10.7], *P*<.21) and LARS categories (Z=–0.264, *P*=.79); however, instant messaging/social media showed a more negative tendency. The kappa values of 3 subjective questions were 0.618, 0.430, and 0.674, respectively. The total LARS scores were consistent between both groups (Pearson coefficient 0.760, *P*<.001; category correlation coefficient 0.570, *P*=.005). Patients with major LARS had highly consistent results, with sensitivity, specificity, kappa value, and *P* value of 77.8%, 91.7%, 0.704, and .001, respectively.

**Conclusions:**

Instant messaging/social media can be a major LARS screening method. However, further research on information accuracy and user acceptance is needed before implementing a mature system.

**Trial Registration:**

ClinicalTrials.gov NCT03009747; https://clinicaltrials.gov/ct2/show/NCT03009747

## Introduction

Low anterior resection syndrome (LARS) is a common functional disorder that develops after patients with rectal cancer undergo anal preservation surgery [1,2]. Its symptoms include changes in defecation frequency, rhythm disorder, incontinence, and constipation, which have been proven to seriously affect the postoperative quality of life [3]. About 30%-55% of patients with rectal cancer have severe LARS symptoms after they complete anal preservation surgery, which can last for several years [4,5].

With improvements in comprehensive treatments for rectal cancer, patients with long-term survival continue to have an increasing demand for LARS treatment, which is a challenge to the current medical system. Its symptoms are variable and persistent, so patients have frequent clinic needs. Common approaches to assess the symptoms of patients with LARS in practice, including face-to-face clinic interviews, post or email questionnaires, and telephone interviews, are often complex and time-consuming, especially when the population of patients with this functional disorder is large [6].

With the popularity of smartphones and mobile internet, remote network technology is changing traditional medical behavior [7,8]. For example, instant messaging/social media represented by WeChat (Tencent Computer System Co., Ltd.) has penetrated Chinese people’s daily life. WeChat has evolved into an information exchange platform widely accepted by people because of its high popularity rate and rich expansibility in China. Medical institutions can develop customized small programs to communicate with patients in batches with the help of many third-party software providers. The positive effects of online follow-up developed based on WeChat platforms in chronic disease prevention and multiple kinds of cancers have been confirmed in several studies [9-14].

Technically, using an instant messaging/social media platform to collect follow-up information has the advantages of privacy, speed, user friendliness, economical value, and fragmented time, which are very suitable for the follow-up needs of patients with LARS. However, an advanced follow-up system based on an instant messaging/social media platform has yet to be made available for clinical use among patients with LARS. To fill this gap, the research team developed an instant messaging/social media follow-up system based on WeChat for patients with LARS and designed a prospective self-controlled clinical study. The team also compared the data from instant messaging/social media and telephone interview and analyzed the consistency of the instant messaging/social media platform.

## Methods

### Study Population

This study was a subproject of the Bas-1611 study. From January 2017 to April 2018, the researchers invited patients who were diagnosed with rectal cancer from 14 medical centers in China and about to receive radical anal preservation surgery. Patients who were histologically proven to have rectal adenocarcinoma 0-12 cm from the anal verge as confirmed by rigid sigmoidoscopy, aged 18 or older, and expected to undergo R0 resection and primary reconstruction were prospectively included. Patients who agreed to participate in the Bas-1611 study and provided informed consent were invited in accordance with the voluntary principle and smartphone usage habits.

### Study Registration

As a subproject of Bas-1611, this study was approved by the ethics committee of the competent authority (2017PHB011-01) and registered on the ClinicalTrials.gov website (NCT03009747). All the enrolled patients received a full informed consent document and signed their consent forms.

### Study Design

#### Development of Questionnaires

A set of questionnaires was established in accordance with the standard of patient-reported outcome measures, including patients’ survival status, defecation satisfaction survey, and Chinese version of the LARS score scale [15]. The questionnaire was transferred into an online version and integrated on the research management platform developed by Servbus Technology Co., Ltd. After signing the informed consent form, the participants scanned the QR code provided by the researcher with their WeChat app and then completed the registration on the platform. The platform automatically sent the questionnaire to the registered WeChat account 3 (90 days), 6 (180 days), and 12 months (365 days) after the operation, and the feedback was saved on the platform database after the participants completed the questionnaire.

#### Telephone Interview

Following the Bas-1611 plan, the participants also received telephone interview at 3 time points. Telephone interview was conducted 1-2 weeks after the WeChat client push. The telephone interview questionnaire was identical to the instant messaging/social media version, but the follow-up personnel would not see the result of the instant messaging/social media follow-up during the interview. All patients enrolled in the study were interviewed by a third-party follow-up team that was employed and trained in a functional follow-up. The interview was properly audio recorded and stored offline to ensure the traceability and quality of research data. The recorded data were rechecked by the experts of the research group against the interview results before data were statistically analyzed to guarantee the accuracy of the telephone interview.

#### Research Questionnaire

Both instant messaging/social media and telephone interview questionnaires contained exactly the same questions and sequences. The following 3 questionnaire forms were used: (1) patient-reported outcome measures (including patients’ survival status and demographic and clinical characteristics); (2) defecation satisfaction survey (including 3 subjective questions Q1, Q2, and Q3); and (3) the Chinese version of the LARS score. The 3 subjective questions included defecation satisfaction, life restriction, and medication use, which required a “yes” or “no” answer. The LARS score was defined as the total score of the items of the questionnaire containing 5 single-choice questions with a corresponding score for each option. Each of the 5 questions tested a single symptom of bowel function, including flatus incontinence, loose stool incontinence, frequency change, clustering, and defecation urgency. The questionnaire could be used to evaluate each patient’s defecation function based on the total score (range 0-42 points), which was divided into 3 categories from best to worst: no LARS (0-20), minor LARS (21-29), and major LARS (30-42) [16].

### Statistical Analysis

Data were collected in pairs for both types at 1 follow-up node and included in statistical analysis. Pearson correlation analysis and paired Student test were conducted for the correlation of continuous variables. Wilcoxon signed rank test was carried out for categorized and ordered variables, and Kendall tau-b correlation analysis was performed for significant correlation. The diagnostic consistency of the kappa value of the instant messaging/social media follow-up method was calculated on the basis of the telephone interview results. *P*<.05 was considered statistically significant. Analyses were performed using SPSS Statistics version 24.0 (IBM Corp).

## Results

### Enrolled Patients, Follow-Up, and Drop-Outs

**Figure 1:**
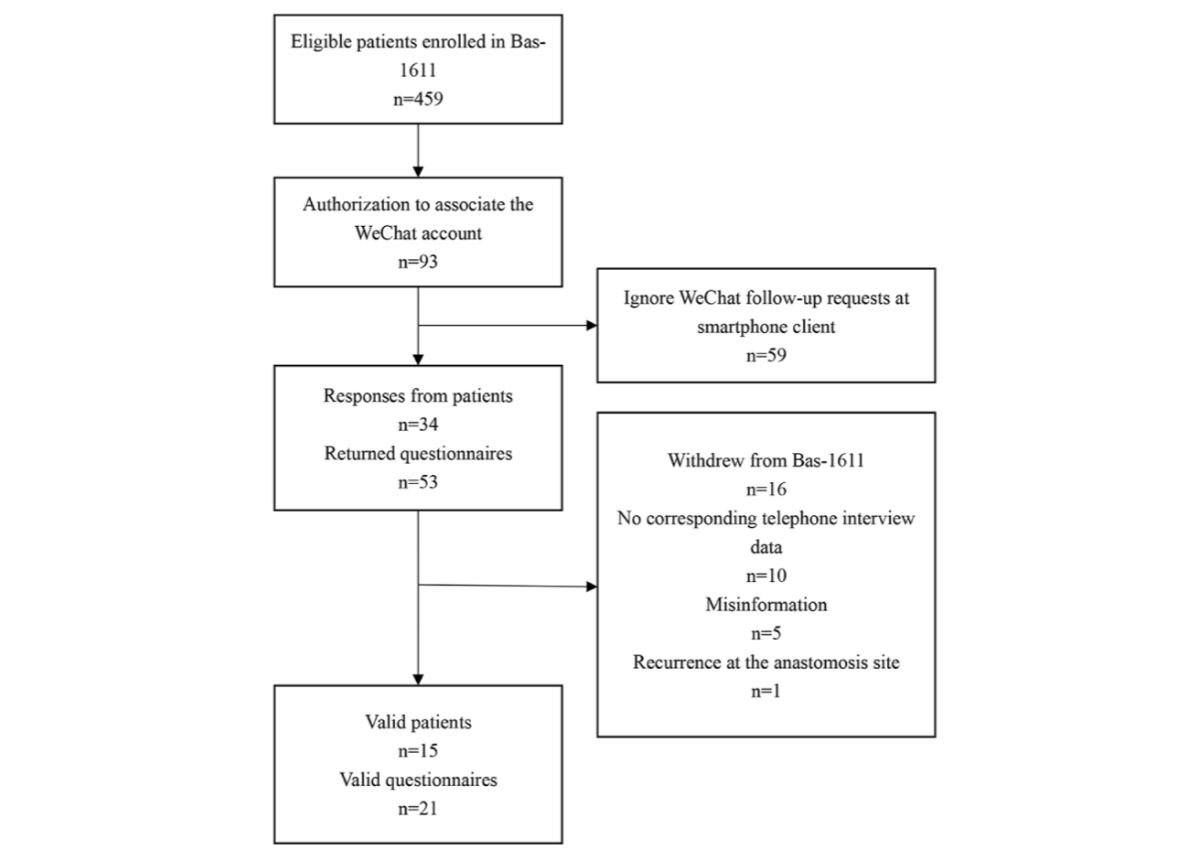
Flowchart of 15 patients.

In total, 459 patients from 14 centers were enrolled in Bas-1611 from January 2017 to April 2019. Among them, 93 patients voluntarily received an additional follow-up via the instant messaging/social media platform and successfully registered a WeChat account. During the study, 212 questionnaires were automatically pushed to these patients via the research management platform. A total of 53 instant messaging/social media questionnaires from 34 patients were collected. Among them, 16 patients dropped out of the Bas-1611 study because of protocol deviation, 10 instant messaging/social media questionnaires had no corresponding telephone interview record, and 5 questionnaires were mistakenly filled before stoma reversal. One patient dropped out because of tumor recurrence. A total of 21 paired instant messaging/social media questionnaires from 15 patients were eventually included in the final analysis (Figure 1).

The 15 patients comprising 10 males and 5 females with an average age of 60.6 (SD 8.3) years were from 4 research centers participating in Bas-1611. A total of 3 patients received preoperative neoadjuvant radiotherapy (cases 3, 5, and 12), while 6 patients received temporary stoma (cases 2, 3, 5, 7, 12, 14) and completed reversion surgery before the expiration date (Table 1).

**Table 1:** Enrolled patient characteristics (N=15).

Variable type and category		Values
**Demographic characteristic**		
	Male, n (%)	10 (67)
	Female, n (%)	5 (33)
	Age at time of surgery (years), mean (SD), range	60.6 (8.3), 49-74
	BMI (kg/m^2^), mean (SD), range	24.5 (1.9), 22.1-27.8
	Distance to anal verge (cm), mean (SD), range	7.3 (2.3), 4-12
**Preoperative TNM classification, n (%)**		
	0	1 (7)
	I	4 (27)
	II	5 (33)
	III	4 (27)
	IV	1 (7)
**Neoadjuvant radiotherapy, n (%)**		
	Yes	3 (20.0)
	No	12 (80.0)
**Diverting stoma, n (%)**		
	Yes	6 (40.0)
	No	9 (60.0)

In Table 2, the answers to the subjective part of 11 questionnaires were completely consistent; 3 questionnaire responses (from case 7, case 13, and case 15) had 2 inconsistent answers, and the remaining 7 questionnaire responses each had 1 inconsistent answer. The results of the telephone interview indicated that 10/21 (48%), 11/21 (52%), and 8/21 (38%) of the participants reported dissatisfaction with their defecation, the effect on the quality of life, and medication use for bowel symptoms, respectively. The corresponding results for the 3 options in the instant messaging/social media follow-up were 10/21 (48%), 13/21 (62%), and 5/21 (24%).

**Table 2:** Response to the 3 subjective questions.

Case number	Time node (months)	Q1^a^			Q2^b^			Q3^c^	
		IMSM^d^	TI^e^	IMSM		TI	IMSM		TI
1	3	Yes	Yes	No		No	No		No
1	6	Yes	Yes	No		No	No		No
2	12	Yes	Yes	No		Yes	No		No
3	12	No	No	Yes		Yes	No		No
4	3	No	No	Yes		Yes	Yes		Yes
5	6	No	No	No		No	No		No
6	6	No	No	Yes		Yes	Yes		Yes
7	6	Yes	Yes	Yes		Yes	No		No
7	12	Yes	No	No		Yes	No		No
8	12	Yes	No	No		No	No		No
9	6	No	No	Yes		Yes	No		No
10	6	Yes	Yes	Yes		No	No		No
11	3	No	No	Yes		Yes	No		Yes
12	6	No	Yes	Yes		Yes	No		No
13	3	No	No	Yes		Yes	Yes		Yes
13	6	No	No	Yes		Yes	No		Yes
13	12	Yes	Yes	Yes		No	No		Yes
14	12	Yes	Yes	No		No	No		No
15	3	Yes	Yes	Yes		No	Yes		Yes
15	6	No	Yes	Yes		No	Yes		Yes
15	12	Yes	Yes	No		No	No		No

^a^Q1: Are you satisfied with the current bowel function?

^b^Q2: Does current defecation affect your daily life?

^c^Q3: Do you use medication to improve your defecation?

^d^IMSM: instant messaging/social media.

^e^TI: telephone interview.

**Figure 2:**
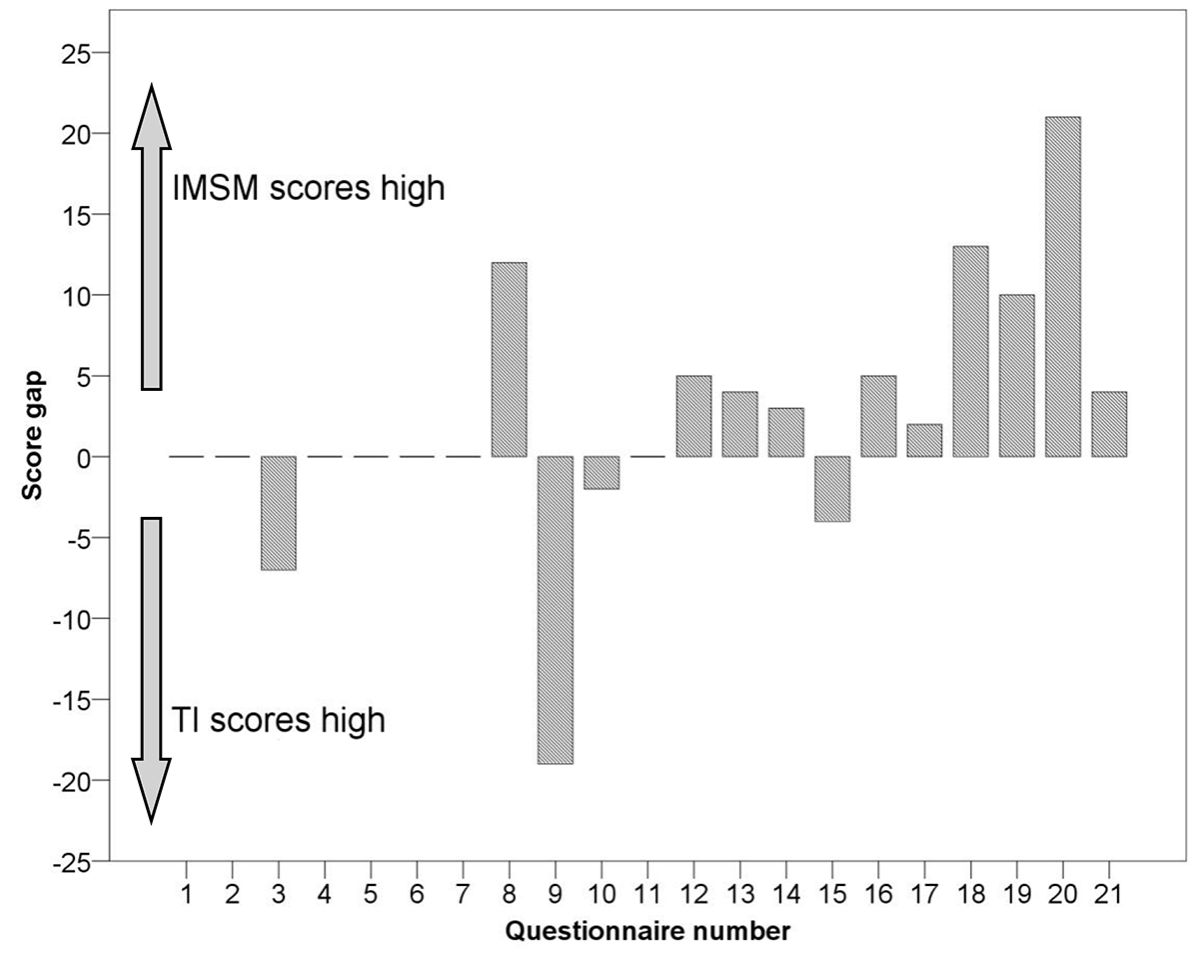
Bar chart of the LARS score difference of 21 paired questionnaires. IMSM: instant messaging/social media; LARS: low anterior resection syndrome; TI: telephone interview.

Multimedia Appendix 1 provides the LARS score results of 21 paired questionnaires. The total score of the LARS questionnaire obtained using the 2 follow-up methods was the same, and ranged from 0 to 39. Although the average score of the instant messaging/social media group was relatively high, the paired *t* test revealed that the 2 methods had no statistically significant difference (22.4 [SD 11.9] versus 24.7 [SD 10.7], t_20_=1.285, *P*=.21). The total score of 10/21 questionnaires (48%) was higher than that of the telephone follow-up, the scores of 7/21 questionnaires (33%) were consistent, and the scores of 4/21 questionnaires (19%) were lower than those of the telephone follow-up (Table 3 and Figure 2).

**Table 3:** Statistical analysis of the LARS score response.^a^

Variables/responses	Score range	Consistency, n	IMSM^b^ score high, n	TI^c^ score high, n	Nonparametric test			Consistency check	
					Wilcoxon *Z* value	*P* value	Kappa value		*P* value
LARS^d^ score	0-42	7	10	4	−1.509	.13	0.760^a^		<.001^a^
LARS category	—	14	4	3	−0.264	.79	0.490		.001
Q1	0-7	10	9	2	−2.032	.04	0.206		.12
Q2	0-3	15	6	0	2.449	.01	0.523		<.001
Q3	0-5	12	0	8	−2.555	.01	0.472		<.001
Q4	0-11	15	4	2	−0.638	.52	0.543		<.001
Q5	0-16	18	1	2	0.001	>.99	0.786		<.001

^a^Because LARS score is a continuous variable, the Pearson coefficient and corresponding *P* value are calculated here.

^b^IMSM: instant messaging/social media.

^c^TI: telephone interview.

^d^LARS: low anterior resection syndrome.

The proportions of no LARS, minor LARS, and major LARS in the telephone interview group were 9, 3, and 9, respectively, whereas their corresponding proportions in the instant messaging/social media interview were 7, 6, and 8. Inconsistencies were observed in the LARS categories of 7 questionnaires, and 2 cases (cases 7 and 15) had extreme inconsistencies. Although the proportion of minor LARS was higher in the instant messaging/social media outcome than in the telephone interview group, the difference was not significant (*Z*=–0.264, *P*=.79).

Each symptom was further analyzed. The instant messaging/social media groups performed worse in 4 symptoms but not in the frequency of bowel movement (Q3). Among them, flatus incontinence (Q1; *P*=.04) and loose stool incontinence (Q2; *P*=.01) showed a statistically significant difference in the Wilcoxon signed rank test. The frequency of bowel movement in the instant messaging/social media questionnaire was significantly better (Q3, *P*=.01). No significant difference was observed in the results of “Q4: clustering”(*P*=.52) and “Q5: defecation urgency”(*P*>.99) Although Q1, Q2, and Q3 were significantly different, the influence on the total score was masked by the high score of Q4 and Q5.

Severe deviations were observed in cases 7 and 15 in the subjective questionnaire and in the LARS score. Case 7, a 56-year-old man with an eye condition, asked his wife to make the choice on the WeChat client for him. Her judgment differed greatly from the patient’s actual feelings, even though they live together. Case 15 was a 50-year-old woman who had repeated episodes of intestinal infection after surgery, which resulted in recurrent diarrhea and incontinence. During the instant messaging/social media follow-up, she described the most recent and severe defecation symptoms, but the symptoms caused by the infection were difficult to be distinguished. Conversely, our telephone interview follow-up staff accurately determined her true defecation condition in the noninfected state.

The patients reported more negative functional results on the instant messaging/social media platform, although no statistically significant difference was obtained due to sample size limitations (Z=–0.264, *P*=.79). For example, 10 (71%) of the 14 questionnaires with inconsistent LARS scores were high, and 4 (67%) of the 6 questionnaires with inconsistent quality of life evaluations chose “yes.” This trend was still evident after cases 7 and 15 were removed.

### Diagnostic Consistency Analysis

The kappa values of the 3 subjective questions were statistically acceptable (Table 4). The sensitivity and specificity of the questionnaire satisfaction survey were 80.0% and 81.8%, respectively, and the diagnostic consistency coefficient was 0.618 (*P*=.006). The results of the quality of life survey were relatively poor; that is, the sensitivity and specificity were 81.8% and 60.0%, respectively. The diagnostic consistency coefficient was 0.430 (*P*=.04). Three questionnaire responses from cases 11 and 13 with differences on medication use were false negative, with a sensitivity of 62.5%, a specificity of 100%, and a diagnostic consistency coefficient of 0.674 (*P*=.001). They were found to have taken an over-the-counter Chinese herbal medicine, which can improve defecation, during the telephone follow-up, and their decision to use medicines was confirmed as a “yes” after the panel discussion.

**Table 4:** Consistency test of the 3 subjective survey results in the instant messaging/social media and telephone interview questionnaires.

Questionnaire	Consistent, n	Inconsistent, n	Sensitivity, %	Specificity, %	Kappa value	*P* value
Q1	17	4	80.0	81.8	0.618	.006
Q2	15	6	81.8	60.0	0.430	.049
Q3	18	3	62.5	100	0.674	.001

The results of the total LARS score were consistent between the 2 groups. The Pearson coefficient was 0.760 (*P*<.001), while the category correlation coefficient was 0.570 (*P*=.005). The kappa value for diagnostic consistency of the LARS category was 0.490 (*P*=.001). Among the 3 categories, the consistency of major LARS was the best, with a sensitivity of 77.8% and a specificity of 91.7% (κ=0.704, *P*=.001). Urgency was the most consistent in diagnosis among all the symptoms (κ=0.786), with only 3 questionnaire responses having different results. Conversely, no statistically significant kappa value was obtained for flatus incontinence (κ=0.206, *P*=.12).

## Discussion

### Principal Results

In this study, a follow-up system for patients with LARS was established on the basis of the instant messaging/social media platform (WeChat app). The results obtained with this method were paired with those obtained with traditional telephone interview. Our findings indicated that the functional outcome of the instant messaging/social media platform was basically consistent with that of telephone interview. In particular, patients with major LARS had a strong consistency and showed more negative functional evaluation trends in the instant messaging/social media platform.

### Application Trend of Instant Messaging/Social Media in Functional Follow-Up

Combined with physical examination and treatment, a face-to-face clinical interview is the most effective way of a functional follow-up [8]. However, it takes time and entails labor costs; furthermore, doctors with LARS management experience are lacking in China. Therefore, LARS management is impossible to be arranged postoperatively for every patient with rectal cancer [17]. In this study, instead of a face-to-face follow-up, telephone interview was used as the control setting, which was also based on the current situation of insufficient outpatient resources for diseases related to defecation function. New methods, such as telephone and remote follow-up, are vital complements to face-to-face follow-up, and their better patient satisfaction and lower cost have been confirmed in the follow-up of patients with cancer and other functional diseases [18,19].

The trend to turn to social media among doctors and patients with cancer for an interchange of disease information is growing [20,21]. However, Pellino et al [20] found that the knowledge acquired by patients with colorectal cancer from open social media is mixed and varied; authoritative arguments are also lacking, while useful knowledge is often overwhelmed by the mass of information. Given the widespread influence of social media, researchers should use it to issue recruitment notices [3,12], but discussing specific symptoms in open social media is difficult, especially for a very private functional disorder such as LARS.

### Consistency of the Instant Messaging/Social Media Platform

Instant messaging/social media has potential as a follow-up platform of patients with LARS because of its privacy, security, convenience, and wide coverage nature, and its accuracy is supported by our research evidence. In this study, responses of patients with major LARS had high consistency, possibly because the symptoms of major LARS are hard to allay. By contrast, patients with mild LARS sometimes had no symptoms; therefore, their feedback fluctuated. This may be because telephone interview was conducted 1-2 weeks after the WeChat client push. The screening and treatment of patients with major LARS are a key part of LARS management, and such patients have difficulty obtaining adequate help from a general oncology client. The stable performance of our follow-up system in major LARS makes it suitable for the follow-up and evaluation of patients with major LARS.

Patients who have cancer and independently complete the questionnaire tend to overstate the extremes on the quality of life [22], especially those whose long-term survival is no longer threatened by cancer. This study explained the higher scores on the instant messaging/social media platform, and they are common in similar studies [23]. The moderate exaggeration of negative feelings in patients is emotionally understandable and may even be common, but whether such exaggeration affects the accuracy of follow-up remains to be further studied.

One of the advantages of LARS scoring is that its logic of question is simple and clear, so patients can easily choose a response [16]. However, such a concise description can likely lead to misunderstanding in the instant messaging/social media platform. In this study, some patients misinterpreted incontinence in flatus and the average number of bowel movement per day. This misunderstanding led to a large discrepancy in Q1 and Q3. A previous study [24] indicated that the LARS questionnaire has defects in evaluating symptoms such as emptying disorder. A functional follow-up on smartphones is a special application scenario in which no professional guidance is available, and appropriate adjustments should be made based on the patient population studied; for example, explanatory words for easily confused parts should be added.

### Patients’ Willingness and Satisfaction

Patients’ willingness to use the instant messaging/social media platform also depends on whether this new method is more convenient and economical than traditional ones. Against the background of generally improved prognosis of colorectal cancer, the medical system is barely being maintained, and providing satisfactory follow-up for outpatient services is difficult. Dai et al [11] found that difficulties in visiting a central medical institution prompt 66.1% of patients to use social media for tracking and feedback. Teagle et al [8] believed that using a remote follow-up technology is an economically feasible solution, which can effectively reduce the burden of the follow-up personnel and reduce the travel cost and missed days of work. Smartphones and instant messaging/social media may be a barrier for some elderly patients, but given that the rectal cancer morbidity has a youth-oriented tendency, this technology may be accepted by more patients in the future. In this study, 10 of the 53 returned questionnaires (19%) were followed up without the corresponding telephone interview, indicating that the convenience of instant messaging/social media might further improve patients’ follow-up intention and response rate in the future.

### Limitations

The main limitation of this study was the low response rate in the instant messaging/social media group, which lead to small sample size in the final analysis. The reasons for low response may be as follows: (1) Elderly patients with rectal cancer were not active users of smartphones; (2) We underestimated the huge information of WeChat app. According to the protocol, we sent only 1 follow-up message at each follow-up node, while the WeChat app may receive dozens or even hundreds of messages every day, due to which some patients failed to notice the follow-up reminder; and (3) The design of the Bas-1611 study made telephone interview available to all patients regardless of their response to the instant messaging/social media follow-up request, possibly resulting in an excessively low WeChat response rate.

Selective bias and outlier results are inevitable because of the small sample size of this study. For the patients to be proficient in using WeChat, younger or better educated patients are needed to be enrolled in studies of this kind. The complexity of instant messaging/social media user behavior leads to the inaccuracy of follow-up information (such as cases 7 and 15), which still needs to be solved in a follow-up study. Inspired by our work, a new randomized controlled trial (BaS-1904, NCT03669237) is committed to further explore the issues of LARS patient management, and it is expected that the aforesaid limitations will also be improved in the new study.

### Conclusions

The instant messaging/social media system provides a promising solution to accommodate the primary follow-up needs of patients with LARS by integrating complex functional follow-up tools into smartphone apps. Although it is currently not a substitute for manual follow-up, it has the potential of becoming a major LARS screening method. However, further research on response rate, information accuracy, and user acceptance is needed before an advanced system can be implemented.

## Appendix

Multimedia Appendix 1 Response of LARS Score.

## Abbreviations

LARS: low anterior resection syndrome
