# Correction: A Novel Remote Follow-Up Tool Based on an Instant Messaging/Social Media App for the Management of Patients With Low Anterior Resection Syndrome: Pilot Prospective Self-Control Study

**DOI:** 10.2196/29325

**Published:** 2021-04-10

**Authors:** Fan Liu, Peng Guo, Xiangqian Su, Ming Cui, Jianlong Jiang, Suo Wang, Zhouman Yu, Runhe Zhou, Yingjiang Ye

**Affiliations:** 1 Department of Gastroenterological Surgery Peking University People's Hospital Beijing China; 2 Beijing Key Laboratory of Colorectal Cancer Diagnosis and Treatment Research Beijing China; 3 Key Laboratory of Carcinogenesis and Translational Research (Ministry of Education), Department of Gastrointestinal Surgery IV Peking University Cancer Hospital and Institute Beijing China; 4 Department of General Surgery Changshu Hospital Affiliated to Soochow University First People's Hospital of Changshu City Changshu China; 5 Department of General Surgery QiLu Hospital (Qingdao) Cheeloo College of Medicine, Shandong University Qingdao China

In “A Novel Remote Follow-Up Tool Based on an Instant Messaging/Social Media App for the Management of Patients With Low Anterior Resection Syndrome: Pilot Prospective Self-Control Study” (JMIR Mhealth Uhealth 2021;9(3):e22647) the authors noted one error.

In the “Acknowledgments” section, a correction has been made to show the correct Grant Number.

The following sentence appeared in the originally published manuscript:

This project is supported by the National Key R&D Program of China (Grant No. 2145000042).

This sentence has been corrected to:

This project is supported by the National Key R&D Program of China (Grant No. 2017YF0908203).

The correction will appear in the online version of the paper on the JMIR Publications website on April 19, 2021, together with the publication of this correction notice. Because this was made after submission to PubMed, PubMed Central, and other full-text repositories, the corrected article has also been resubmitted to those repositories.

