# Supplementary material for: A Novel Remote Follow-Up Tool Based on an Instant Messaging/Social Media App for the Management of Patients With Low Anterior Resection Syndrome: Pilot Prospective Self-Control Study
Source: JMIR Mhealth Uhealth. 2021 Mar 19;9(3):e22647. doi: 10.2196/22647 (PMC8078008; doi:10.2196/22647)
Supplement: Multimedia Appendix 1 [file mhealth_v9i3e22647_app1.docx]

Appendix 1. Response of LARS Score.^a^

| No. | Patient | Time node | Q1^b^ | | Q2^c^ | | Q3^d^ | | Q4^e^ | | Q5^f^ | | Total score | | LARS score Category | |
| --- | --- | --- | --- | --- | --- | --- | --- | --- | --- | --- | --- | --- | --- | --- | --- | --- |
|  |  |  | IMSM | TI | IMSM | TI | IMSM | TI | IMSM | TI | IMSM | TI | IMSM | TI | IMSM | TI |
| 1 | Case 1 | 3 months | 0 | 0 | 0 | 0 | 0 | 0 | 9 | 9 | 0 | 0 | 9 | 9 | No LARS | No LARS |
| 2 | Case 1 | 6 months | 0 | 0 | 0 | 0 | 2 | 2 | 9 | 9 | 0 | 0 | 11 | 11 | No LARS | No LARS |
| 3 | Case 2 | 12 months | 4 | 7 | 0 | 0 | 0 | 4 | 11 | 11 | 0 | 0 | 15 | 22 | No LARS | Miner LARS |
| 4 | Case 3 | 12 months | 7 | 7 | 3^h^ | 3^h^ | 2 | 2 | 9 | 9 | 11 | 11 | 32 | 32 | Major LARS | Major LARS |
| 5 | Case 4 | 3 months | 4 | 4 | 3^h^ | 3^h^ | 4 | 4 | 11 | 11 | 16 | 16 | 38 | 38 | Major LARS | Major LARS |
| 6 | Case 5 | 6 months | 0 | 0 | 3^g^ | 3^g^ | 0 | 0 | 11 | 11 | 16 | 16 | 30 | 30 | Major LARS | Major LARS |
| 7 | Case 6 | 6 months | 7 | 7 | 3^g^ | 3^g^ | 5 | 5 | 0 | 0 | 0 | 0 | 15 | 15 | No LARS | No LARS |
| 8 | Case 7 | 6 months | 7 | 4 | 0 | 0 | 0 | 0 | 9 | 0 | 11 | 11 | 27 | 15 | Miner LARS | No LARS |
| 9 | Case 7 | 12 months | 0 | 7 | 0 | 0 | 0 | 4 | 9 | 11 | 11 | 16 | 20 | 32 | No LARS | Major LARS |
| 10 | Case 8 | 12 months | 0 | 0 | 0 | 0 | 0 | 2 | 0 | 0 | 0 | 0 | 0 | 2 | No LARS | No LARS |
| 11 | Case 9 | 6 months | 7 | 7 | 3^g^ | 3^g^ | 2 | 2 | 11 | 11 | 16 | 16 | 39 | 39 | Major LARS | Major LARS |
| 12 | Case 10 | 6 months | 7 | 0 | 0 | 0 | 0 | 2 | 11 | 11 | 11 | 11 | 29 | 24 | Miner LARS | Miner LARS |
| 13 | Case 11 | 3 months | 4 | 0 | 3^h^ | 0 | 2 | 5 | 11 | 11 | 16 | 16 | 36 | 32 | Major LARS | Major LARS |
| 14 | Case 12 | 6 months | 7 | 0 | 3^g^ | 0 | 2 | 4 | 11 | 11 | 11 | 16 | 34 | 31 | Major LARS | Major LARS |
| 15 | Case 13 | 3 months | 4 | 0 | 3^g^ | 0 | 4 | 4 | 0 | 11 | 16 | 16 | 27 | 31 | Miner LARS | Major LARS |
| 16 | Case 13 | 6 months | 4 | 0 | 3^g^ | 0 | 2 | 4 | 11 | 11 | 16 | 16 | 36 | 31 | Major LARS | Major LARS |
| 17 | Case 13 | 12 months | 0 | 0 | 0 | 0 | 2 | 2 | 11 | 9 | 16 | 16 | 29 | 27 | Miner LARS | Miner LARS |
| 18 | Case 14 | 12 months | 4 | 0 | 0 | 0 | 0 | 0 | 9 | 0 | 0 | 0 | 11 | 0 | No LARS | No LARS |
| 19 | Case 15 | 3 months | 7 | 0 | 3^g^ | 0 | 2 | 2 | 11 | 11 | 0 | 0 | 23 | 2 | Miner LARS | No LARS |
| 20 | Case 15 | 6 months | 7 | 0 | 3^g^ | 0 | 2 | 2 | 9 | 9 | 11 | 0 | 32 | 11 | Major LARS | No LARS |
| 21 | Case 15 | 12 months | 0 | 0 | 3^g^ | 3^g^ | 0 | 5 | 9 | 0 | 11 | 11 | 23 | 19 | Miner LARS | No LARS |

^a^Each number represents the score corresponding to the patient's answer.

^b^Q1: Do you ever have occasions when you cannot control your flatus (wind)?

^c^Q2: Do you ever have any accidental leakage of liquid stool? (The two Q2 options differ in degree, but are assigned the same value of 3.)

^d^Q3: How often do you open your bowels?

^e^Q4: Do you ever have to open your bowels again within one hour of the last bowel opening?

^f^Q5: Do you ever have such a strong urge to open your bowels that you have to rush to the toilet?

^g^Yes, less than once per week

^h^Yes, at least once per week.
